# Celiac Disease Monocytes Induce a Barrier Defect in Intestinal Epithelial Cells

**DOI:** 10.3390/ijms20225597

**Published:** 2019-11-09

**Authors:** Deborah Delbue, Danielle Cardoso-Silva, Federica Branchi, Alice Itzlinger, Marilena Letizia, Britta Siegmund, Michael Schumann

**Affiliations:** Department of Gastroenterology, Infectious Diseases and Rheumatology, Campus Benjamin Franklin, Charité – Universitätsmedizin Berlin, 12203 Berlin, Germany; deborah.delbue-da-silva@charite.de (D.D.); danielle.cardoso-da-silva@charite.de (D.C.-S.); federica.branchi@charite.de (F.B.); alice.itzlinger@charite.de (A.I.); marilena.letizia@charite.de (M.L.); britta.siegmund@charite.de (B.S.)

**Keywords:** barrier function, tight junction assembly, monocytes, celiac disease

## Abstract

Intestinal epithelial barrier function in celiac disease (CeD) patients is altered. However, the mechanism underlying this effect is not fully understood. The aim of the current study was to evaluate the role of monocytes in eliciting the epithelial barrier defect in CeD. For this purpose, human monocytes were isolated from peripheral blood mononuclear cells (PBMCs) from active and inactive CeD patients and healthy controls. PBMCs were sorted for expression of CD14 and co-cultured with intestinal epithelial cells (IECs, Caco2BBe). Barrier function, as well as tight junctional alterations, were determined. Monocytes were characterized by profiling of cytokines and surface marker expression. Transepithelial resistance was found to be decreased only in IECs that had been exposed to celiac monocytes. In line with this, tight junctional alterations were found by confocal laser scanning microscopy and Western blotting of ZO-1, occludin, and claudin-5. Analysis of cytokine concentrations in monocyte supernatants revealed higher expression of interleukin-6 and MCP-1 in celiac monocytes. However, surface marker expression, as analyzed by FACS analysis after immunostaining, did not reveal significant alterations in celiac monocytes. In conclusion, CeD peripheral monocytes reveal an intrinsically elevated pro-inflammatory cytokine pattern that is associated with the potential of peripheral monocytes to affect barrier function by altering TJ composition.

## 1. Introduction

Celiac disease (CeD) is an autoimmune enteropathy triggered by the ingestion of gluten, affecting approximately 1% of the population in Western countries [1]. Current understanding of CeD immune pathology focusses on activation of gluten-specific TH1-cells secondary to presentation of DQ2- or DQ8-restricted gliadin peptides as the cause of the small intestinal inflammatory response. As a consequence, inflammation leads to villous atrophy and crypt hyperplasia, thereby causing the typical clinical features of intestinal malabsorption of nutrients [2,3].

A so-far unresolved issue of CeD is the nature of the associated intestinal epithelial barrier defect. It not only occurs secondary to the inflammatory process located in the lamina propria in active disease, but appears to be primary, since it is verifiable in inactive CeD patients on a gluten-free diet (GFD) and in relatives of CeD patients who do not suffer from CeD [4,5]. Moreover, Kumar et al. identified barrier-defining genes associated with CeD, thereby providing genetic proof for the relevance of barrier function in CeD pathogenesis. Importantly, the barrier function is maintained by a protein-protein complex interaction, where the main structure is the tight junction (TJ) proteins. TJs are the most apical contact between enterocytes formed by integral membrane proteins, including occludin, claudins, and scaffolding proteins as ZO-1 [6]. Although structural changes in celiac barrier function can be allocated to enterocyte TJ composition and epithelial transcytosis of gliadin peptides, research aiming to clarify how these changes arise is scarce [7,8,9,10].

Interestingly, monocytes are strongly involved in the regulation of intestinal barrier function, either by secretion of cytokines or by direct interaction with intestinal epithelial cells [11,12,13]. After extravasation, monocytes infiltrate the lamina propria, differentiating into macrophages and producing inflammatory mediators to combat pathogens [14]. For CeD, it has been previously described that monocytes isolated from CeD patients produced substantial amounts of TNF-α and interleukin-8 (IL-8) in a gluten-dependent manner [15]. Moreover, a gliadin-stimulated monocytic cell line was shown to have the potential to modulate intestinal epithelial barrier function [16]. Furthermore, monocytes isolated from healthy individuals that were stimulated with IL-15 as a celiac-mimicking cytokine milieu were capable of secreting pro-inflammatory cytokines that are known to induce barrier defects [17].

In the current work, we aimed to analyze the potency of celiac monocytes to perturb intestinal barrier function. For this purpose, we isolated monocytes from CeD patients and co-cultured these cells with intestinal epithelial cells to analyze epithelial barrier function.

## 2. Results

### 2.1. Monocytes Derived from Celiac Patients Induce a Barrier Defect in Intestinal Epithelial Cells

**Figure 1:**
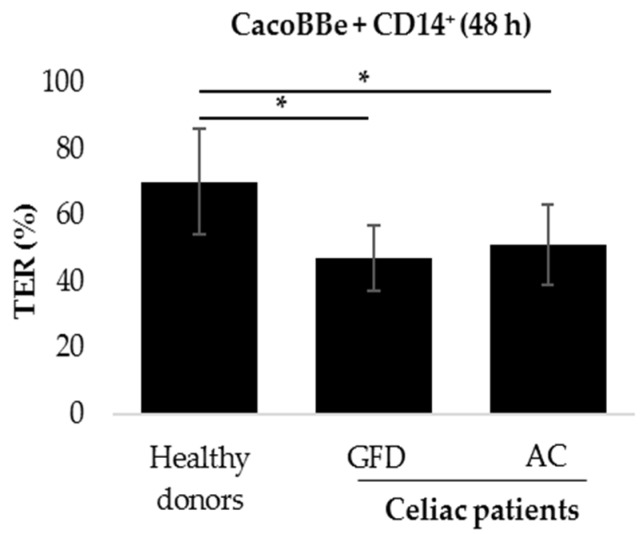
Epithelial barrier function after co-culture of intestinal epithelial cells (IECs) with monocytes derived from celiac disease patients. After peripheral blood mononuclear cell (PBMC) isolation and CD14+ cell-sorting, epithelial cells were co-cultured with monocytes from healthy donors. celiac patients on a gluten-free diet (GFD), or AC (active celiac disease (CeD)) patients. Subsequently, the TER was measured after 48 h of co-culture (% of TER prior to addition of monocytes). Mean of *n* = 36 (healthy donors), *n* = 15 (GFD), and *n* = 20 (AC) individual filters measurements. Monocytes used for these experiments were isolated from *n* = 8 (healthy donors), *n* = 4 (GFD) and *n* = 5 (active CeD). Mann-Whitney U * *p* < 0.05, comparison between co-cultures with monocytes from healthy donors and CeD patients.

To evaluate whether monocytes exert an effect on epithelial barrier function, intestinal epithelial cells (IECs) were co-cultured with human primary monocytes. Peripheral blood mononuclear cells (PBMCs) were isolated from peripheral blood of CeD patients with different disease status and of healthy donors and then sorted for CD14 expression. IECs were grown on transwell filters, where they reached confluence and built up a stable barrier function, whereas the monocytes were placed underneath the filters. Thus, interaction between monocytes and IECs was possible mostly through soluble factors that pass through the filter membranes. Interestingly, transepithelial resistance (TER) of IECs measured after 48 h of co-culture was reduced when they were co-cultured with celiac monocytes, compared to the co-culture with healthy controls. This effect added up to approx. 70% of the control level and was irrespective of CeD activity (Figure 1).

Co-culturing IECs with unsorted (i.e., total) PBMCs caused a similar decrease in TER (Figure S1). To exclude possible direct effects of gliadin or IL-15 on the epithelium, CacoBBe cells were exposed to IL-15/Tglia alone, with monocytes or PBMCs. In IECs alone, we did not observe a decrease in TER with only IL-15/Tglia addition. Nevertheless, in the cells exposed to monocytes, TER decreased at the same levels as with monocytes plus IL-15/Tglia. (Figure S2). These results showed that effects observed in TER are independent of IL-15/Tglia stimulation and that this is rather directly associated with monocytes. In summary, this experiment uncovered the potential of celiac monocytes to alter epithelial barrier function.

### 2.2. Celiac Monocytes Alter IEC-TJ Structure

**Figure 2:**
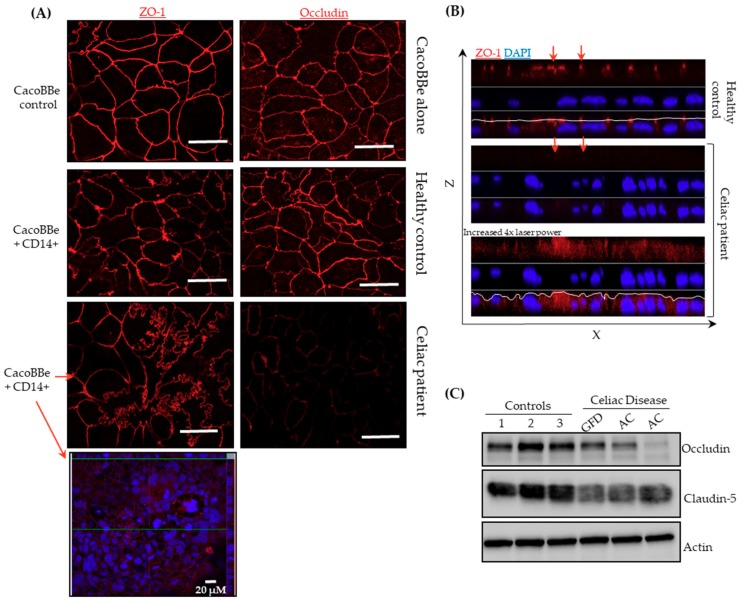
Tight junction (TJ) structure and protein composition after co-culture with monocytes derived from celiac disease patients. (**A**) Cellular localization of occludin and ZO-1 were investigated using confocal laser scanning microscopy after immunostaining. Representative images from *n* = 5 (healthy donors), *n* = 3 (CeD on GFD) and *n* = 3 (Active CeD patients). Scale bar: 50 µM. The two effects of CD14 co-culture in ZO-1 expression is pointed out by red arrows (**B**) Collapsed XZ-projections. ZO-1 staining reveals apical junctional complexes at approx. identical Z heights, as illustrated by the white lining in the merged image. When CacoBBe cells co-cultured with celiac monocytes were immunostained, lining was comparably irregular, and ZO-1 level was reduced. (**C**) IEC protein levels by Western blotting of occludin and claudin-5 after co-culture with monocytes.

As a next step, we aimed to evaluate whether CD14^+^ cells alter IEC barrier function through changes in tight junction (TJ) integrity. First, IECs that had completed 48 h of co-culture with CD14^+^ monocytes were immunostained for TJ proteins ZO-1 and occludin. As shown in Figure 2, no effect was found regarding TJ localization or expression in IEC layers that had been co-cultured with CD14^+^ monocytes isolated from healthy donors. However, for IECs co-cultured with monocytes derived from CeD patients, lower levels of occludin and a mosaic expression pattern of ZO-1 was found, with ZO-1 being significantly reduced in expression in some, but not all, regions of the filter. Moreover, TJs appeared to be irregular regarding loop-like linings, which was not observed when IECs were exposed to monocytes of healthy donors (Figure 2A). Additionally to the aforementioned reduction in expression level of ZO-1, XZ-projections revealed an uneven structure of the apical membrane in IECs that were co-cultured with CeD monocytes (Figure 2B). Furthermore, protein levels of occludin and the TJ-sealing claudin-5, which has previously been implicated in the CeD barrier defect, were analyzed (Figure 2C) [9]. IEC protein levels of occludin and claudin-5, after exposure to celiac monocytes, were reduced compared to protein levels of the respective healthy control monocytes. These data show evidence that CeD monocytes exert effects on the TJ structure of co-cultured IECs.

### 2.3. Monocytes Derived from Celiac Disease Patients Present Higher Levels of Proinflammatory Cytokine Production

**Figure 3:**
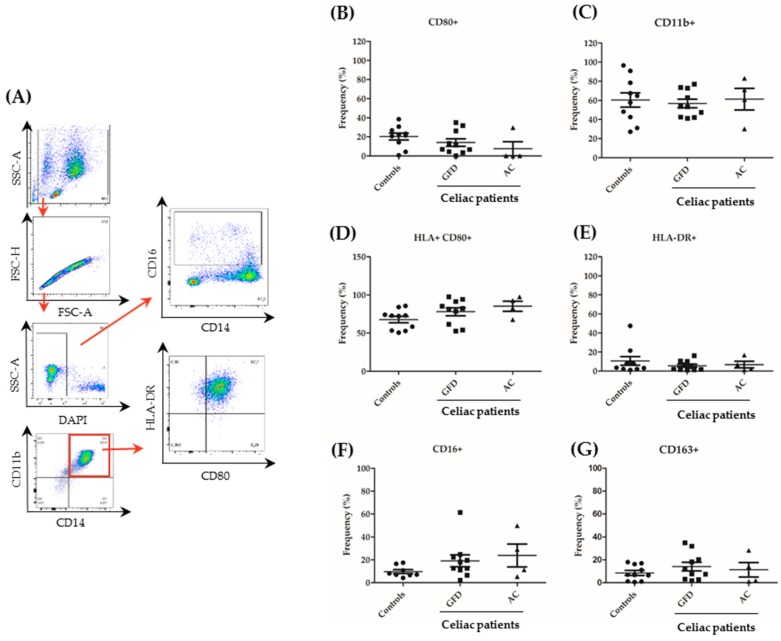
Expression of surface markers in peripheral monocytes from celiac patients. PBMCs were isolated from 10 controls, 10 GFD and 4 active CeD (AC) patients and sorted for CD14. Subsequently, cells were cultured in the presence of granulocyte macrophage colony stimulating factor (GM-CSF; 10 ng/ml) for 24 h and evaluated by flow cytometry. (**A**) Gating strategy used to determine surface marker expression. The stepwise gating approach is highlighted by various steps of analysis that are interconnected by red arrows. Representative plots from a healthy control are shown. (**B**–**G**) Results for the expression of single surface markers are shown. Each dot represents the surface marker expression result of a single patient. Mean values ± standard error of the mean (SEM) are shown. The Mann–Whitney U test revealed no significant differences.

Next, we characterized isolated human monocytes that had previously been sorted for CD14 to uncover potential differences regarding cytokine and surface marker expression between celiac and healthy control monocytes. First, we analyzed the expression of surface markers that are characteristic of classically and non-classically activated macrophages. Surface marker expression was analyzed after CD14-sorting (Figure S3) and 24 h of culture—media including Granulocyte macrophage colony stimulating factor (GM-CSF)—by immunostaining. Using a gating strategy revealed in Figure 3A, monocyte populations were detected, doublets were excluded, and the viable population (DAPI-negative cells) was analyzed. Then, frequency of positivity for CD11b, CD80, HLA-DR, CD163, and CD16 was evaluated. No significant differences in the frequency of any of the examined surface markers were found (Figure 3B-G; Figure S3). The frequency of cells revealing a double positivity for CD80 and HLA-DR (i.e., expression of both inflammatory markers within the same cell) was also analyzed but turned out to be not significantly different. Although CD16 frequency was not significantly increased, a tendency toward higher frequencies of CD16-positive cells was observed. This points to an increased fraction of intermediate/non-classical monocytes in the peripheral blood of CeD patients.

**Figure 4:**
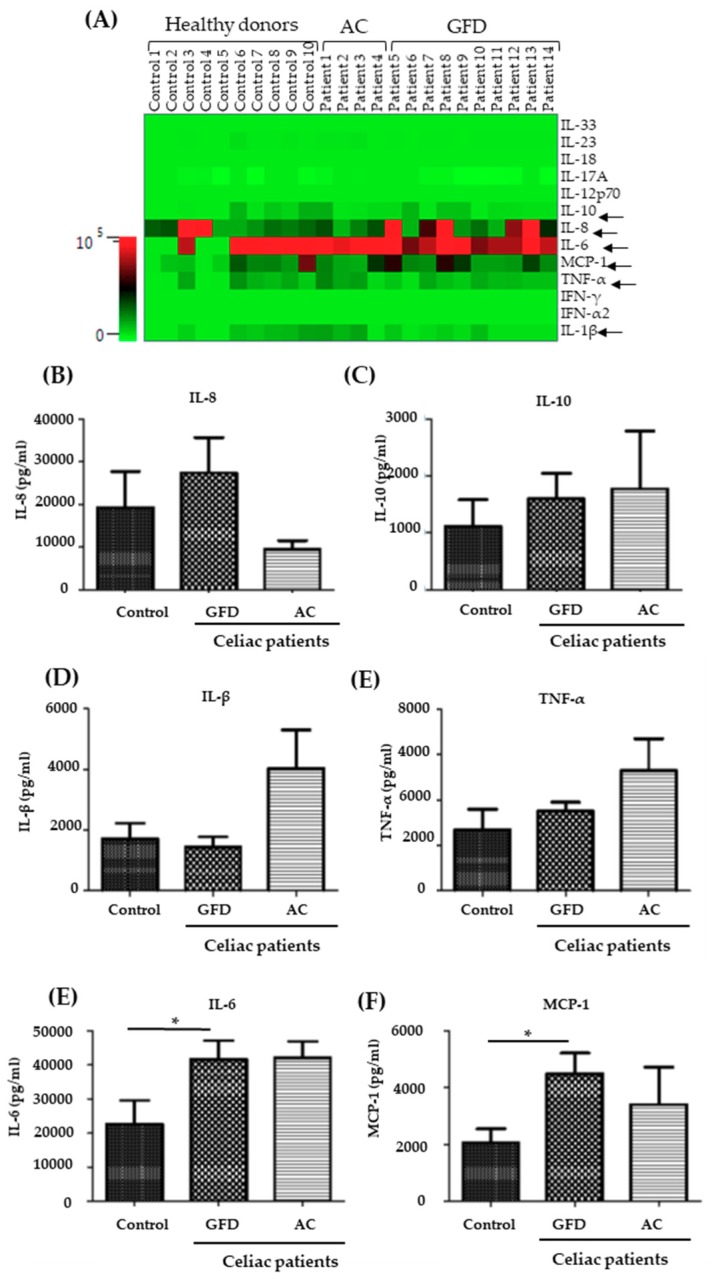
Increased levels of pro-inflammatory cytokines in the supernatant of monocytes derived from celiac disease patients. (**A**) PBMCs were isolated from 10 controls, 10 GFD, and 4 active CeD patients, and monocytes were sorted using CD14 MACS MicroBeads. Subsequently, cells were kept in the incubator for 24 h in the presence of GM-CSF (10 ng/mL). Supernatants of monocyte cultures were collected and cytokine levels were determined. Data are illustrated as a heat map revealing color-coded concentrations of cytokines (green: low concentration; red: high concentration). (**B**–**F**) Results for individual cytokine measurements are shown: Interleukin- (IL-)8, IL-10, IL-1β, TNF-α, IL-6, and MCP-1. Mean values ± SEM are shown. Mann–Whitney test: *, *p < 0.05*.

Subsequently, we determined the concentration of a set of cytokines within the supernatant of the monocytes at the end of the 24 h time period following isolation and CD14-sorting. To guarantee survival, they were cultured at this time in the presence of GM-CSF (10 mg/ml). As illustrated in Figure 4A, concentrations of IFN-α2, IFN-λ, IL-19, IL-12p70, IL-17A, IL-18, IL-23, and IL-33 did not reach the detection level of the assay, neither in the control nor the CeD group. More interestingly, levels of IL-1β, TNF-α, IL-8, IL-10, IL-6, and MCP-1 were found at detectable levels. Although concentrations of IL-1β, TNF-α, IL-8, and IL-10 were not significantly different between the groups of patients and healthy controls, a tendency for higher levels of the pro-inflammatory cytokines was observed in CeD supernatants (Figure 4B–E). Interestingly, IL-6 and MCP-1 (monocyte chemotactic protein-1, synonymous: CC-chemokine ligand-2, CCL2) were found at significantly higher levels in the supernatant of monocytes from CeD patients who had received a GFD, compared to monocytes from healthy controls (Figure 4E,F). In summary, these data reveal that CD14^+^ monocytes isolated from CeD patients carry a more pro-inflammatory phenotype, an effect that appears to be independent of disease activity.

## 3. Discussion

CeD is an autoimmune enteropathy triggered by the ingestion of dietary gluten [3]. Although the central role for on the one hand gluten-specific, and on the other hand tissue-resident, cytotoxic T-cells is undisputed, results from genome-wide association studies and from functional data collected in non-affected relatives of CeD patients point to a primary defect of the epithelial barrier as a discrete pathophysiologic entity within the overall immune pathology of CeD [2,5,18]. Nevertheless, it is mostly unclear how the barrier defect in CeD is triggered. Since monocytes are major mediators in mucosal barrier defects, we aimed to elucidate the potential of peripheral human monocytes to alter intestinal epithelial barrier function. The rationale for using peripheral blood monocytes comes from early data that convincingly showed that in intestinal inflammation, monocytes infiltrate the mucosa and then further differentiate to macrophages secreting pro-inflammatory cytokines, rather than showing that resident tissue-macrophages re-differentiate into pro-inflammatory macrophages [19].

CD14^+^ monocytes derived from peripheral blood of CeD patients revealed an intrinsically higher secretion of IL-6 and MCP-1. Moreover, a non-significant tendency for increased expression of TNF-α and IL-1β was observed. A similar pattern of cytokine expression was previously described for intestinal (i.e., not peripheral) macrophages in IBD [20,21]. Specifically, Kamada et al. described the CD14+ macrophage population secondary to their cytokine expression (e.g., TNF-α, IL-6, IL-12/23p40, and IL-23p19) as pro-inflammatory and non-resident, compared to CD14^-^ resident macrophages that are found primarily in healthy gut and do not express pro-inflammatory cytokines. Phenotypically similar macrophages were shown to elicit a barrier defect on IECs in a co-culture model, similar to that used in the current work [11]. However, in the current work, expression analysis of IL-1β and TNF-α, which were previously shown by Lissner et al. to be mostly responsible for the IEC barrier defect in the M1- and M0-polarized macrophage model, only revealed a non-significant tendency towards higher levels of these cytokines. On the other hand, IL-6 and MCP-1 were significantly increased. MCP-1 (synonymous CCL-2) is secreted by various cells, including monocytes, as a chemoattractant for monocytes, T-cells, and dendritic cells, and enables via the CCL2-CCR2 axis the extravasation of monocytes into the lamina propria [14,22]. Our data on MCP-1 are in line with data from Italy that revealed a higher expression of CCL-2 by PBMCs after stimulation with the p31-43 gliadin peptide [23]. IL-6, a pro-inflammatory cytokine also secreted by intestinal macrophages, has previously been described to induce an intestinal barrier defect by increasing expression of pore-forming claudins, including claudin-2 [24]. Interestingly, IL-6 secreted by macrophages as the cause for a reduced intestinal barrier function was also discussed as a mechanism for the epithelial barrier defect found in liver cirrhosis [25].

Although a difference in cytokine production was found between celiac monocytes and monocytes isolated from healthy controls, which was in line with the functional barrier data revealing a defect only inducible by peripheral monocytes from CeD patients, we did not observe significant differences in monocyte surface marker expression. CD16 as a marker for alternatively activated macrophages showed a non-significant tendency towards higher expression in celiac monocytes. However, this aspect of our work remains somewhat inconclusive.

Monocytes isolated from CeD patients induced a functional barrier defect on IECs. The reduced barrier function was measured by TER and was associated with an altered TJ morphology on confocal laser scanning microscopy (LSM) as well as TJ protein expression in Western blotting. Celiac barrier defects that are localized to the TJ have been described previously [9,26]. In those studies, the alteration of the celiac TJ was complex, involving reduced expression of occludin, claudin-3, -5, and -7, and altered phosphorylation of ZO-1. This is mostly in line with our work, since celiac monocytes induced a reduction of occludin and claudin-5 expression, and confocal LSM revealed alterations in cellular ZO-1 distribution. Nevertheless, we should mention that we and others have shown that apoptosis of IECs, which was not analyzed in the current study, might also contribute to a defective epithelial barrier and that induction of (apoptotic or non-apoptotic) cell death might be a conceivable fate of an IEC that is exposed to monocytes [9,27].

Taken together, our results suggest that in CeD patients, peripheral blood monocytes have the potential to induce an epithelial defect of the intestinal mucosa. This IEC reaction to monocyte exposure is presumably related to action of monocytic IL-6 on IECs. Tight junctional alterations in the intestinal epithelial cell layer are at least partially responsible for the functional barrier defect. These effects might be primary in nature. However, work herein is not sufficient to prove the primary nature. In the future, this could be approached by analyzing monocytes of first-degree relatives to CeD patients to determine if these cells also show an impact on barrier function.

## 4. Materials and Methods

### 4.1. Human Material

The study was approved by the Ethics Committees of the Charité – Universitätsmedizin Berlin, Germany (protocol number EA4/116/18, accepted on Jan 22nd, 2019). Heparinized whole blood samples were collected from healthy individuals and CeD patients. Inactive (GFD) patients received a GFD for >1 year. All patients declared their informed consent (signed consent form). Healthy controls were individuals without a history of enteropathy and without clinical signals of CeD or other autoimmune diseases. For further characteristics of CeD patients, refer to Table 1.

### 4.2. Cell Line

The human colorectal cell line, CacoBBe (C2BBe1 [clone of Caco-2] ATCC® CRL-2102™), was maintained in Dulbecco’s Modified Eagle’s Medium (DMEM) + GlutaMAX (Gibco), with 10% fetal bovine serum (Gibco), 1% penicillin and streptomycin (Corning), 10µM HEPES-buffer and 1M non-essential amino acids (Merck Millipore). Cells were kept at 37 °C in a 5% CO_2_ environment. Culture medium was changed three times per week.

### 4.3. PBMCs Isolation and CD14+ Sorting

Peripheral blood mononuclear cells (PBMCs) were isolated from peripheral blood of healthy donors and celiac disease patients by Biocoll (Merk Millipore) separating solution and centrifugation, as previously described [28]. Subsequently, PBMCs were sorted using CD14 MACS MicroBeads (Miltenyi Biotech, Bergisch Gladbach, Germany). As determined by flow cytometry, preparations contained >90% CD14+ cells. Monocytes were plated in 24-well dishes with RPMI-1640 as media (Gibco), supplemented with 10% of fetal bovine serum (Gibco) and 1% of penicillin and streptomycin (Corning). CD14+ cells received human granulocyte macrophage colony stimulating factor (10ng/mL; GM-CSF) for 24 h prior to co-culture. Cell culture supernatants were collected for flow cytometry analysis.

### 4.4. Co-Culture and TER Measurement

Intestinal epithelial cells (IECs) were plated on permeable transwell polycarbonate filter supports (0.4 µm; 0.6 cm^2^, Merck Millipore) and kept at 37 °C in a 5% CO_2_ environment. Culture medium was changed three times per week. On days 10 to 16 after plating, filters were transferred to 24-well dishes containing CD14^+^ cells (5 × 10^5^ cells per well). In addition, IL-15/Tglia (10 mg/mL) was added (gift by W. Dieterich; LPS-free) to filters with IECs. Subsequently, the transepithelial resistance (TER) was measured, as described previously [11], for 48 h of co-culture with monocytes from CeD patients or healthy donors (controls). For immunofluorescence experiments, filters were fixed using 1% paraformaldehyde for 15 minutes at room temperature.

### 4.5. Immunofluorescence

Epithelial cell layers were stained using the following primary antibodies: ZO-1 (1:100; BD Biosciences, NJ, USA). The secondary antibodies used were Alexa Fluor 488 goat anti-mouse or rabbit IgG, and Alexa Fluor 594 goat anti-mouse or rabbit IgG (1:500; Thermo Fisher Scientific, MA, USA). To determine occludin expression and cellular distribution, an occludin mouse monoclonal antibody (OC-3F10) was used as an Alexa Fluor® 594 Conjugate (Thermofischer). Nuclei were stained using DAPI (4′,6-Diamidin-2-phenylindol, conc. 1:2000). Immunofluorescence staining was analyzed by confocal laser scanning microscopy (LSM 780, Carl Zeiss, Jena, Germany) as previously described [9,11].

### 4.6. Western Blotting

For Western blotting analysis, the protocol was followed as previously described [9]. The primary antibodies used were occludin (1:1000; Sigma Aldrich, St. Louis, MO, USA), claudin-5 (1:1000; Invitrogen, Carlsbad, CA, USA) and actin (1:1000; Sigma Aldrich, St. Louis, MO, USA). The peroxidase-conjugated secondary antibodies used were goat anti-rabbit IgG or goat anti-mouse IgG (Jackson ImmunoResearch, Ely, UK). SuperSignal West Pico PLUS Stable Peroxide Solution (Thermo Scientific, Waltham, MA, USA) was used for protein detection, and Fusion FX7 imaging system (Vilber Lourmat Deutschland GmbH, Eberhardzell, Germany) was used to detect protein signal levels.

### 4.7. Flow Cytometric Assessment—Surface Markers and Cytokine Expression

CD14^+^ cells were washed twice with PBS, and the surface markers were checked. The following fluorochrome-coupled antibodies were applied: anti-CD14 (61D3) from BD Biosciences, anti- anti-CD16 (3G8), anti-CD11b (ICRF44), anti-CD163 (GHI/61) from Biolegend, CD80 (2D10.4), and anti-HLA-DR (LN3) from eBioscence. Dead cells were excluded by DAPI staining. Samples were assessed by flow cytometry using a FACSCanto II and the FACS Diva software (version 6; BD Biosciences). Supernatants of the cultures after 24 h of culture with GM-CSF (10 ng/ml) were tested for cytokine expression (IL-1β, IFN-α2, IFN-λ, TNF-α, MCP-1, IL-6, IL-8, IL-19, IL-12p70, IL-17A, IL-18, IL-23, and IL-33) using the LEGENDplex Multi-Analyte Flow Assay kit–Human Inflammation Panel (13-plex) (Biolegend) according to the manufacturer’s protocol. FACS data were analyzed using FlowJo (v10.6.1) and LEGENDplex v8.0 software (BioLegend, San Diego, CA, USA).

### 4.8. Statistical Analysis

Statistical analysis was performed using GraphPad Prism software (GraphPad Software, La Jolla, CA) by a non-parametric Mann–Whitney test to analyze differences between the control and the CeD patients. One-way ANOVA was used to compare differences in all groups analyzed. All data are expressed as mean values ± standard error of the mean (SEM). A *p <* 0.05 was considered significant.

## Figures and Tables

**Table 1:** Characteristics of CeD patients.

Number of Subjects	17
Female/Male	14/3
Age at enrolment, median (range)	46 (23-83)
Age at CeD diagnosis, median (range)	32 (6-73)
**Marsh Grade at enrolment**, *n* (%)	
0	5 (29)
1	2 (12)
2	0 (0)
3a	1 (6)
3b	3 (18)
3c	0 (0)
not available	6 (35)
**tTG at enrolment**, *n* (%)	
positive	6 (35)
negative	2 (12)
not available	9 (53)
**HLA-DQ status**, *n* (%)	
DQ2+	11 (65)
DQ8+	0 (0)
not available	6 (35)
**GFD status**, *n* (%)	
Active CeD	6 (35)
New CeD diagnosis	4
CeD, non-compliant to GFD	2
CeD on GFD	11 (65)

CeD: celiac disease; tTG: transglutaminase antibodies; GFD: gluten-free diet.

## Supplementary Materials

Supplementary materials can be found at https://www.mdpi.com/1422-0067/20/22/5597/s1.

## Abbreviations

AC	Active celiac
CeD	Celiac disease
CCL2	CC-chemokine ligand-2
GFD	Gluten-free diet
GM-CSF	Granulocyte macrophage colony stimulating factor
IL-1β	Interleukin-1β
IL-6	Interleukin-6
IL-8	Interleukin-8
IL-10	Interleukin-10
IL-12p70	Interleukin-12p70
IL-15	Interleukin-15
IL-17A	Interleukin-17A
IL-18	Interleukin-18
IL-23	Interleukin-23
IL-33	Interleukin-33
IFN-γ	Interferon-γ
IFNα2	Interferon-α2
MACS	Magnetic cell sorting
MCP-1	Monocyte chemotactic protein-1
PBMCs	Peripheral blood mononuclear colony stimulating factor
TER	Transepithelial resistance
Tglia	Trypsinized gliadin
TJ	Tight junctions
