# Supplementary material for: Celiac Disease Monocytes Induce a Barrier Defect in Intestinal Epithelial Cells
Source: Int J Mol Sci. 2019 Nov 9;20(22):5597. doi: 10.3390/ijms20225597 (PMC6888450; doi:10.3390/ijms20225597)
Supplement: Supplementary file 1 [file ijms-20-05597-s001.pdf]

International Journal of Molecular Sciences

Supplementary material

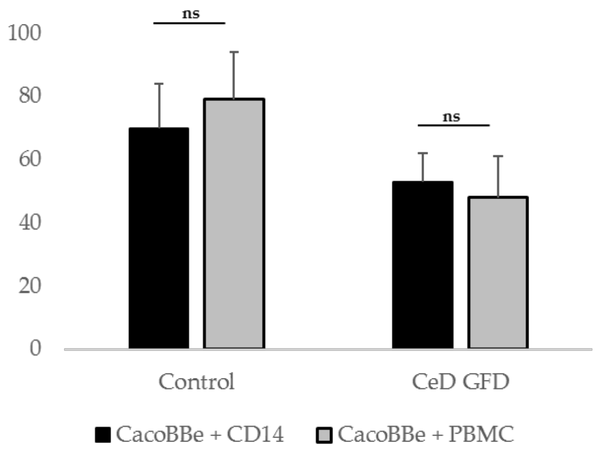

Figure S1: Comparison of the effects on epithelial integrity after co-culture with total PBMCs and CD14+ cells. After PBMC isolation and CD14+ cell-sorting, epithelial cells were co-cultured with monocytes or total PBMCs from healthy donors or celiac patient on GFD (CeD GFD). Subsequently, TER was measured after 48 h of co-culture. Mean of n=8 (healthy donors), n=8 (GFD) individual filters measurements. Monocytes used for these experiments were isolated from n=2 (healthy donors) and n=2 (GFD). Mann-Whitney U, comparison between co-cultures with monocytes and total PBMCs from healthy donors and CeD patients. ns: non-significant.

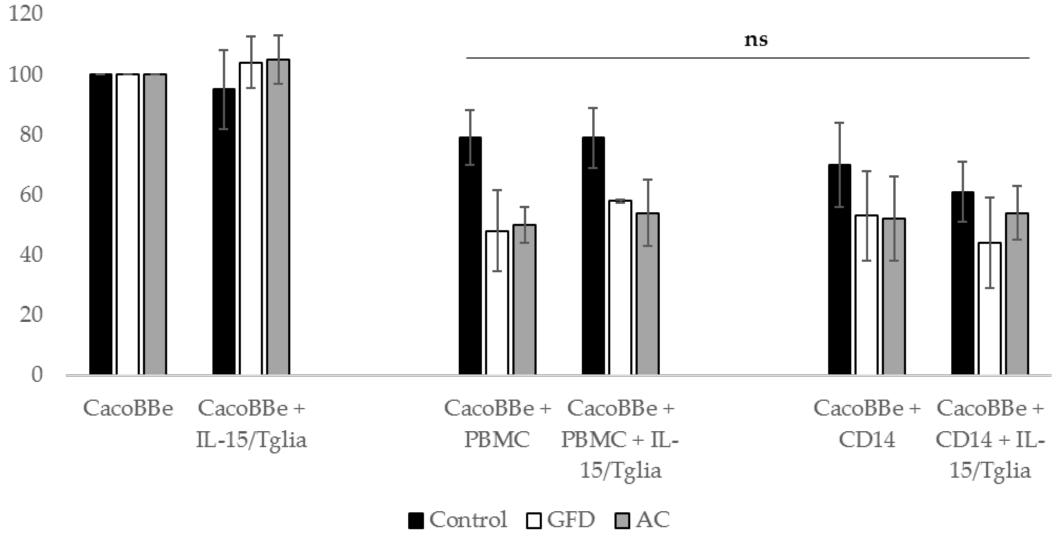

Figure S2: Comparison of the effects on epithelial integrity after addition of IL-15/Tglia on CacoBBe cells with or without monocytes or PBMCs exposure. Intestinal epithelial cells were co-cultured with monocytes or total PBMCs from healthy donors or celiac patient on GFD or AC (active CeD patients). Subsequently, the TER was measured after 48 h of co-culture (% of TER prior to addition of monocytes). Mean of n=36 (healthy donors), n=15 (GFD) and n=20 (AC) individual filters measurements. Monocytes used for these experiments were isolated from n=8 (healthy donors), n=4 (GFD) and n=5 (active CeD). Mann-Whitney U; non-significant.

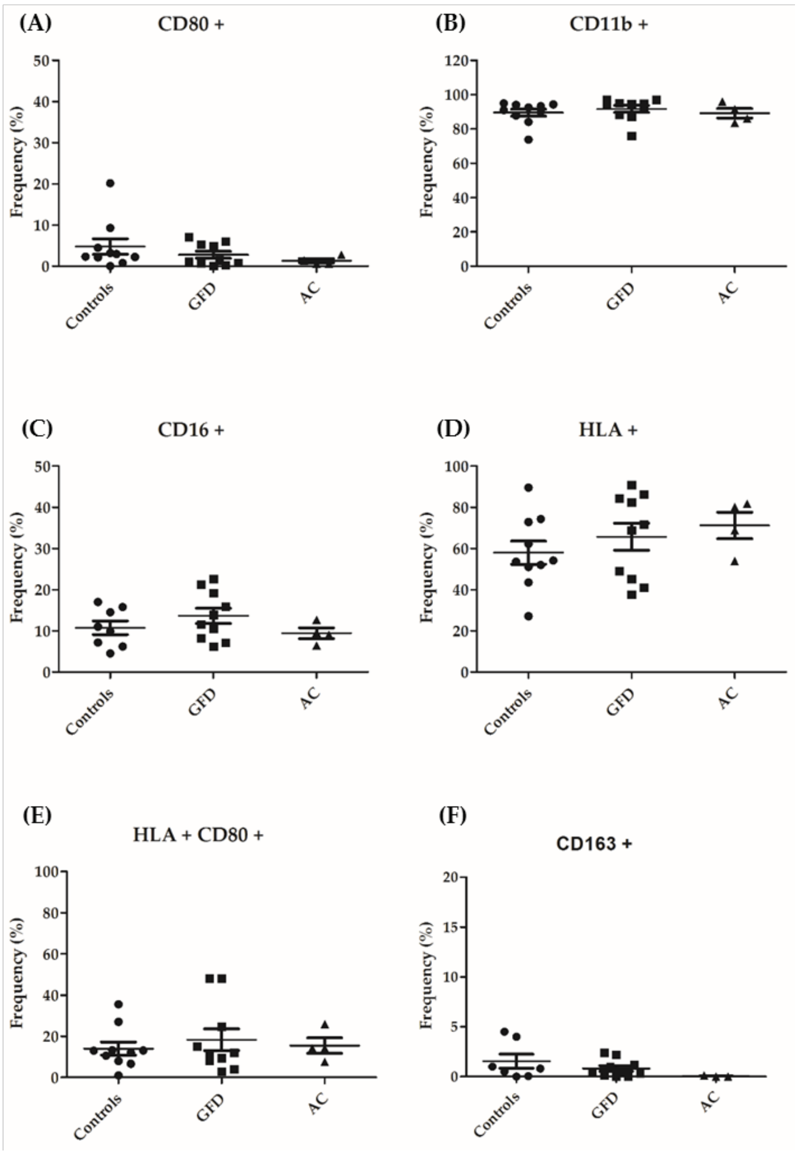

Figure S3. Expression of surface markers in peripheral monocytes from celiac patients after CD14+ sorting. PBMCs were sorted for CD14 and evaluated by flow cytometry. (A) to (F) Expression of surface markers are shown. Each dot represents the expression of a surface marker in a single patient. Mean values ± SEM are shown. Mann Whitney U test revealed no significant differences.
